# Inhibition of the pentose phosphate pathway by dichloroacetate unravels a missing link between aerobic glycolysis and cancer cell proliferation

**DOI:** 10.18632/oncotarget.6272

**Published:** 2015-11-02

**Authors:** Géraldine De Preter, Marie-Aline Neveu, Pierre Danhier, Lucie Brisson, Valéry L. Payen, Paolo E. Porporato, Bénédicte F. Jordan, Pierre Sonveaux, Bernard Gallez

**Affiliations:** ^1^ Louvain Drug Research Institute (LDRI), Biomedical Magnetic Resonance Research Group, Université Catholique de Louvain (UCL), Brussels, Belgium; ^2^ Institut de Recherche Expérimentale et Clinique (IREC), Pole of Pharmacology, Université Catholique de Louvain (UCL), Brussels, Belgium

**Keywords:** bioenergetics, glycolysis, dichloroacetate, pentose phosphate pathway, proliferation

## Abstract

Glucose fermentation through glycolysis even in the presence of oxygen (Warburg effect) is a common feature of cancer cells increasingly considered as an enticing target in clinical development. This study aimed to analyze the link between metabolism, energy stores and proliferation rates in cancer cells. We found that cell proliferation, evaluated by DNA synthesis quantification, is correlated to glycolytic efficiency in six cancer cell lines as well as in isogenic cancer cell lines. To further investigate the link between glycolysis and proliferation, a pharmacological inhibitior of the pentose phosphate pathway (PPP) was used. We demonstrated that reduction of PPP activity decreases cancer cells proliferation, with a profound effect in Warburg-phenotype cancer cells. The crucial role of the PPP in sustaining cancer cells proliferation was confirmed using siRNAs against glucose-6-phosphate dehydrogenase, the first and rate-limiting enzyme of the PPP. In addition, we found that dichloroacetate (DCA), a new clinically tested compound, induced a switch of glycolytic cancer cells to a more oxidative phenotype and decreased proliferation. By demonstrating that DCA decreased the activity of the PPP, we provide a new mechanism by which DCA controls cancer cells proliferation.

## INTRODUCTION

These last few years, metabolism has generated tremendous interest in the field of cancer research. Many studies focused on the various metabolic profiles of different tumors [1–3] because metabolic plasticity is involved in cancer progression, drug resistance and metastasis [4–6]. In normal cells, glycolysis is coupled to oxidative phosphorylation (OXPHOS) to optimally synthesize intracellular ATP from glucose [7]. However, many cancer cells undergo a fundamental metabolic transformation, the “glycolytic switch”, by which glycolysis is uncoupled from respiration and rewired to lactic fermentation, thus becoming the primary source of cell ATP production. Switching to a glycolytic metabolism primarily occurs under hypoxia as a rescue mechanism for energy production. However, some cancer cells further adopt a particular glycolytic phenotype, first described by Warburg [8] and coined ‘aerobic glycolysis’ [9]. The biological rationale behind the Warburg phenotype remains controversial, but it has been recently proposed that proliferating cancer cells enhance glycolysis because it benefits both bioenergetics and biosynthesis [4, 10]. Indeed, a glycolytic metabolism potentially allows fast ATP production and provides carbon intermediates that can be directed to branched biosynthetic pathways, enabling a faster expansion of the cellular biomass. Convincingly, mutations occurring in signaling pathways regulating both cellular biosynthesis and aerobic glycolysis, such as the PI3K/Akt/mTOR pathway, are the most prevalent class of mutations in human tumors [11]. However, experimental evidence linking aerobic glycolysis to cancer cell proliferation is lacking, and the selective advantage provided by this phenotype is not entirely clear. The aim of the present study was to elucidate the coupling between metabolism, energy supply and cell proliferation in various human and murine cancer cells. Metabolic switches were induced to provide evidence that bioenergetics, and more particularly glycolysis, directly drives cancer cell proliferation. In this line, we found a new therapeutic mechanism of dichloroacetate (DCA), an activator of the mitochondrial oxidation of glucose currently investigated in clinical studies [12]. DCA inhibited the pentose phosphate pathway (PPP), a pivotal biosynthetic pathway branched to glycolysis. We report that the PPP bridges the gap between a glycolytic metabolism and cancer cell proliferation.

## RESULTS

### Glycolytic efficiency is positively linked to proliferation but not to ATP levels in cancer cells

**Figure 1:**
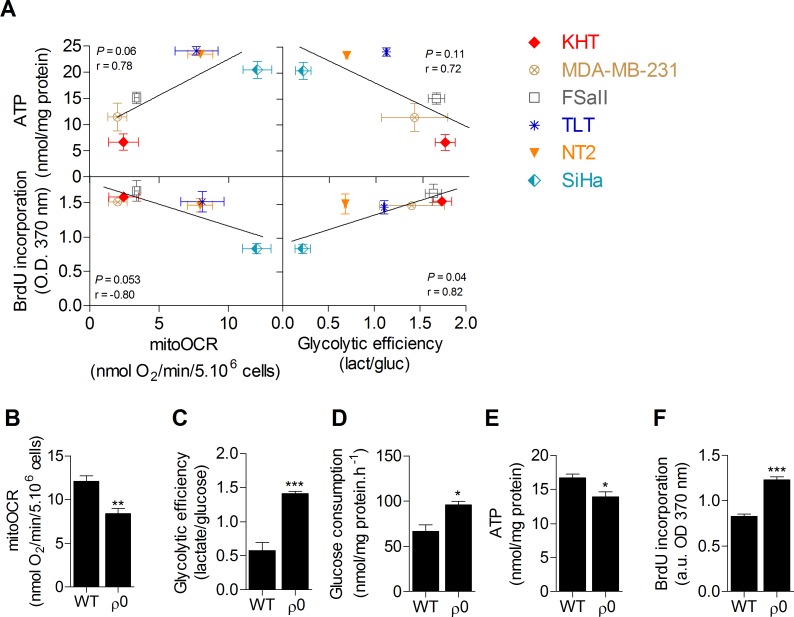
Glycolytic efficiency is positively linked to proliferation but not to ATP levels in cancer cells **A.** Correlation plots between metabolic parameters (glycolytic efficiency and mitochondrial oxygen consumption rate [mitoOCR]), intracellular ATP content and proliferation of six human and murine cancer cell lines. Measurements were performed after 24 h incubation in the presence of a culture medium containing only glucose as energetic fuel. The mitoOCR was determined by the oligomycin-sensitive OCR of viable whole cells and the glycolytic efficiency (glucose consumption/lactate production ratio) was measured from cells supernatant. Total ATP was quantified from lysed cells and normalized to protein content. Proliferation rates were analyzed by the incorporation of a nucleotid analog (5-bromo-2′-deoxyuridine [BrdU]). A significant correlation was found between glycolytic efficiency and proliferation (*p*-value = 0.04, Pearson *r* = 0.82). Non-significant correlations were found between mitoOCR and ATP content (*p*-value = 0.06, Pearson *r* = 0.78), mitoOCR and proliferation (*p*-value = 0.053, Pearson *r* = −0.80) and between glycolytic efficiency and ATP content (*p*-value = 0.11, Pearson *r* = 0.72). **B.**-**F.** Comparison of the metabolic profile (B-C-D), intracellular ATP content **E.** and proliferation **F.** between wild-type (WT) and mitochondria-depleted (ρ0) isogenic SiHa cancer cells. Two-sided *t* test. *p < 0.05, **p < 0.01, ***p < 0.001. Results are expressed as means ± SEM.

A screening of the metabolic activities of six cancer cell lines was first performed in order to investigate the link between glucose metabolism, ATP stores and proliferation capacity. OXPHOS, evaluated by the oligomycine-sensitive oxygen consumption rate (mitoOCR), and glycolytic efficiency, measured by the lactate produced to glucose consumed (mol/mol) ratio [13], were assessed separately on cells incubated 24 hours in a culture medium containing only glucose as energetic fuel. To investigate the influence of the metabolic status on cellular energy stores, total intracellular ATP pool was also measured. No significant correlation was found between intracellular ATP content and the metabolic parameters. However, we found that cell proliferation evaluated by DNA synthesis was significantly correlated to glycolytic efficiency (Figure 1A), where increased glycolysis was associated with an increased proliferation capacity, and inversely. No significant correlation was observed between proliferation capacity and mitochondrial respiration. These data suggest that glycolysis is potentially the key energetic pathway supporting cancer cell proliferation. It is currently hypothesized that this pathway supports cell proliferation predominantly by supplying precursors for biosynthetic pathways, rather than by ATP production [10, 11]. Supporting that ATP supply is not the main limiting factor for cell proliferation, no correlation between ATP content and DNA synthesis was found in the cell lines (Supplemental Figure S1). To confirm that glycolysis promotes cancer cell proliferation and exclude metabolism-independent influences inherent to different genotypes, the proliferation capacity of human wild-type (WT) oxidative SiHa cervical cancer cells was compared to that of SiHa with partial mitochondrial depletion (SiHa ρ0) [14, 15]. SiHa ρ0 cancer cells exhibited a glycolytic phenotype with a ~40% decrease in mitoOCR (Figure 1B), a ~2.5 fold increase in glycolytic efficiency (Figure 1C) and a ~50% increase in glucose consumption (Figure 1D). The net ~20% decrease in total ATP pool in SiHa ρ0 cells confirmed that enhanced glycolysis was not sufficient to maintain ATP stores (Figure 1E). By performing DNA synthesis quantification, we found that enhanced aerobic glycolysis promotes cell proliferation, as the glycolytic switch in SiHa ρ0 cells was associated with a ~45% increase in cell proliferation (Figure 1F). Viability assays revealed no difference in viable cell number 24 h after cells were incubated in the experimental culture medium (Supplemental Figure S2), likely because DNA synthesis quantification detects early changes in the proliferation rate of the cells.

### Glycolysis inhibition by DCA impairs cancer cell proliferation

**Figure 2:**
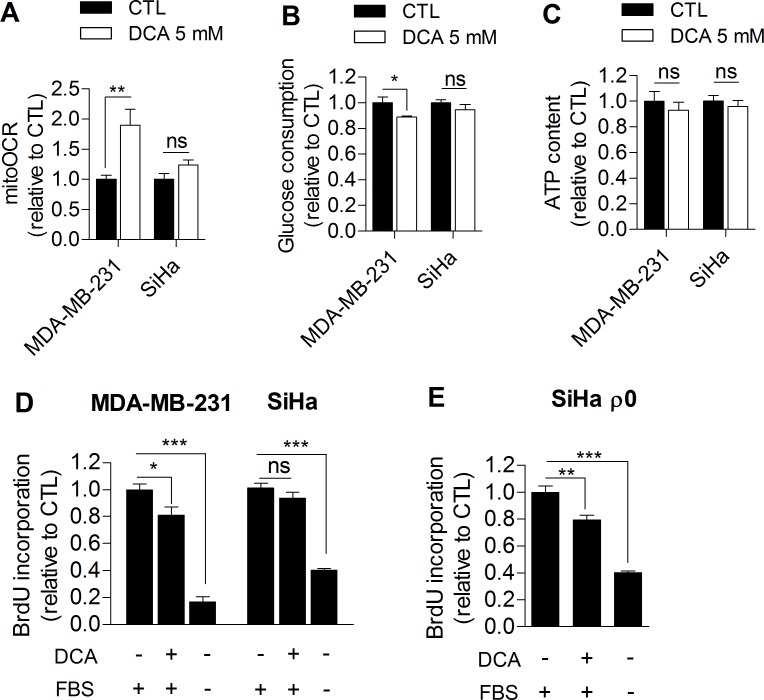
DCA significantly influences the metabolism and proliferation of glycolytic but not oxidative cancer cells **A.** Mitochondrial oxygen consumption rate, **B.** glucose consumption, **C.** intracellular ATP content and **D.** proliferation of MDA-MB-231 (glycolytic) and SiHa (oxidative) human cancer cells after 48 h dichloroacetate (DCA) 5 mM treatment. **E.** Proliferation of mitochondria-depleted (ρ0) SiHa cancer cells after 48 hours DCA 5 mM treatment. Medium containing no FBS was used as positive control in proliferation experiments. Two-sided *t* test **A.**-**C.** or one-way ANOVA with Bonferroni post-hoc test **D.**-**E.** *p < 0.05, **p < 0.01, ***p < 0.001, ns, not significant. Results are expressed as the relative change from control cells and as means ± SEM.

Based on our observations, we further investigated whether glycolysis inhibition could directly impair cancer cell proliferation. For the purpose, MDA-MB-231 human breast cancer cells (Warburg phenotype, Figure 1A) and SiHa human cervical cancer cells (oxidative phenotype, Figure 1A) were treated with dichloroacetate (DCA), a pyruvate dehydrogenase kinase (PDK) inhibitor that enhances the oxidative activity of cells by activating pyruvate dehydrogenase (PDH), the gate-keeping enzyme of glucose oxidation in mitochondria [16]. To date, the promising therapeutic effect of DCA on cancer cells is globally attributed to a normalization of the hyperpolarized mitochondrial membrane potential characterizing cancer cells and to re-sensitization to apoptosis [17]. Here, we postulated that DCA also controls tumor proliferation by inhibiting glycolysis. To test this hypothesis, glycolytic MDA-MB-231 and oxidative SiHa cancer cells were treated with 5 mM DCA for 48 h, and the effects of the treatment on metabolism and proliferation were assessed. Compared to vehicle-treated cells, DCA induced a switch of glycolytic MDA-MB-231 cancer cells to a more oxidative phenotype as evidenced by an increase in mitoOCR (Figure 2A) and a decrease in glucose consumption (Figure 2B). The decrease in glycolytic activity observed in this experiment is consistent with another recent study [18] and is likely induced by the Pasteur Effect [4, 19] to maintain ATP homeostasis in the cells (Figure 2C). We also observed that glycolysis inhibition by DCA was associated with a decreased proliferation rate of MDA-MB-231 cancer cells (Figure 2D). Supporting that glycolysis inhibition impairs proliferation in this cell line, 2-Deoxy-D-glucose-treated MDA-MB-231 cancer cells also exhibited a decreased proliferation rate (Supplemental Figure S3).

On the other hand, DCA did not significantly alter the metabolic activities of oxidative SiHa cancer cells (Figure 2A-2C) and had no significant effects on SiHa proliferation (Figure 2D). Short-term (1 hour) lactate production measurements showed that DCA was indeed more effective in the glycolytic cancer cell line than in the oxidative one (Supplemental Figure S4). To investigate whether the metabolic profile determine the response to DCA, the proliferation capacity of glycolytic SiHa ρ0 cancer cells was also analyzed after DCA treatment. We found a significant decrease in DNA synthesis in this cell line (Figure 2E), an effect that was not observed in SiHa WT. Furthermore, the same number of viable MDA-MB-231 and SiHa ρ0 cancer cells were measured 48 h after treatment with vehicle or DCA (Supplemental Figure S5 A-B), showing that the effects of DCA on metabolic functions and proliferation rates are not due to cell mortality.

### Glycolysis controls cancer cell proliferation through the pentose phosphate pathway

**Figure 3:**
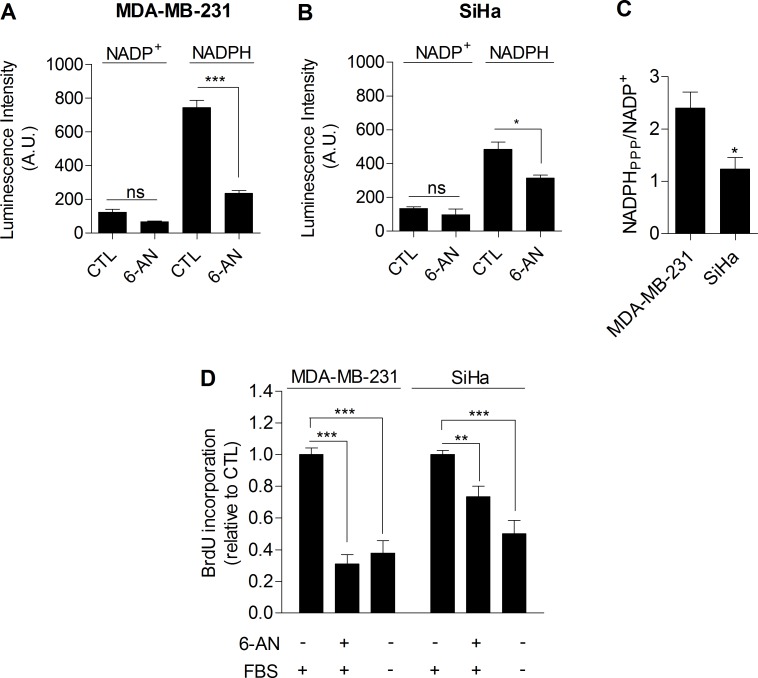
The PPP differentially supports proliferation in glycolytic and oxidative cancer cells Intracellular NADP^+^ and NADPH levels measured individually in viable glycolytic MDA-MB-231 **A.** and oxidative SiHa **B.** cancer cells. 6-aminonicotinamide (6-AN), a specific inhibitor of the PPP, was used (100 μM, 48 h treatment) to highlight NADPH production from the PPP (NADPH_ppp_). **C.** NADPH_PPP_/NADP^+^ ratios in MDA-MB-231 and SiHa cancer cells. **D.** Proliferation measured by the incorporation of BrdU in MDA-MB-231 and SiHa cancer cells after exposure to 6-AN (100 μM, 48 h treatment). Medium containing no FBS was used as positive control in proliferation experiments. Two-sided *t* test **A.**-**C.** or one-way ANOVA with Bonferroni post-hoc test **D.**. *p < 0.05, **p < 0.01, ***p < 0.001, ns, not significant. Results are expressed as means ± SEM.

**Figure 4:**
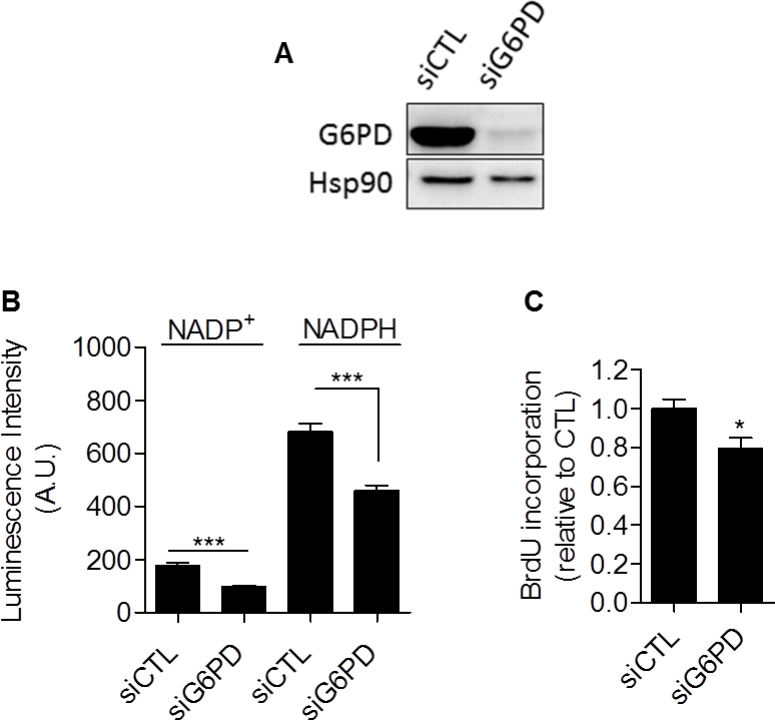
Glucose-6-phosphate dehydrogenase inhibition with siRNA reduces proliferation of glycolytic cancer cells MDA-MB-231 cancer cells were transfected with siRNAs targeting G6PD (siG6PD) or non-targeting siRNAs (siCTL). 48 hours after transfection, cells were subjected to **A.** immunoblot analysis using Hsp90 as loading control, **B.** NADP^+^ and NADPH levels quantification and **C.** DNA synthesis measurement. Two-sided *t* test. *p < 0.05, ***p < 0.001. Results are expressed as means ± SEM.

Taken together, our data provided compiling experimental evidence that glycolysis *per se* is involved in the control of cell proliferation. The mechanism linking glycolysis and proliferation still remained to be established. We postulated that the pentose phosphate pathway (PPP) could link glycolysis to proliferation, as the PPP uses glycolytic intermediates to supply cells with nucleotides and NADPH, a crucial reductant in anabolic processes [20]. To specifically determine NADPH produced from the PPP (NADPH_ppp_), cells were treated with 6-aminonicotinamide (6-AN), a specific inhibitor of the PPP [21, 22]. We found that the contribution of NADPH_ppp_ to the total NADPH pool (NADPH_tot_) was more predominant in MDA-MB-231 cancer cells than in SiHa cancer cells, as evidenced by a major decrease in NADPH_tot_ level following 6-AN treatment in glycolytic MDA-MB-231 cells (Figure 3A). More limited effects were seen in SiHa oxidative cells (Figure 3B). Measurements also revealed higher NADPH_ppp_/NADP^+^ ratio in MDA-MB-231 cancer cells, highlighting a higher PPP flux in this glycolytic cell line compared to SiHa (Figure 3C). To verify that the PPP is involved in the control of cancer cells proliferation, DNA synthesis in 6-AN-treated and non-treated cells was evaluated. We observed that the proliferation capacity of cancer cells was impaired when the PPP was inhibited (Figure 3D). Interestingly, a stronger effect was evidenced in glycolytic MDA-MB-231 (~70 % decrease in DNA synthesis rate) compared to oxidative SiHa (~25 % decrease in DNA synthesis rate) cancer cells (Figure 3D). Importantly, SiHa ρ0 were more sensitive than SiHa WT to 6-AN as a ~40 % decrease in DNA synthesis was found (Supplemental Figure S6). Owing to the potential off-target effects of pharmacological inhibitors, we complemented our 6-AN studies using small interfering RNAs (siRNAs) targeting glucose-6-phosphate dehydrogenase (G6PD), the first and rate-limiting enzyme of the PPP [23]. In MDA-MB-231 transfected cells, we confirmed silencing of G6PD by immunoblotting (Figure 4A) and demonstrated that, like 6-AN, inhibition of G6PD decreased PPP activity (Figure 4B) and DNA synthesis (Figure 4C). These results point out the predominant contribution of the PPP in sustaining the proliferation of Warburg-phenotype cancer cells.

### DCA inhibits the pentose phosphate pathway

**Figure 5:**
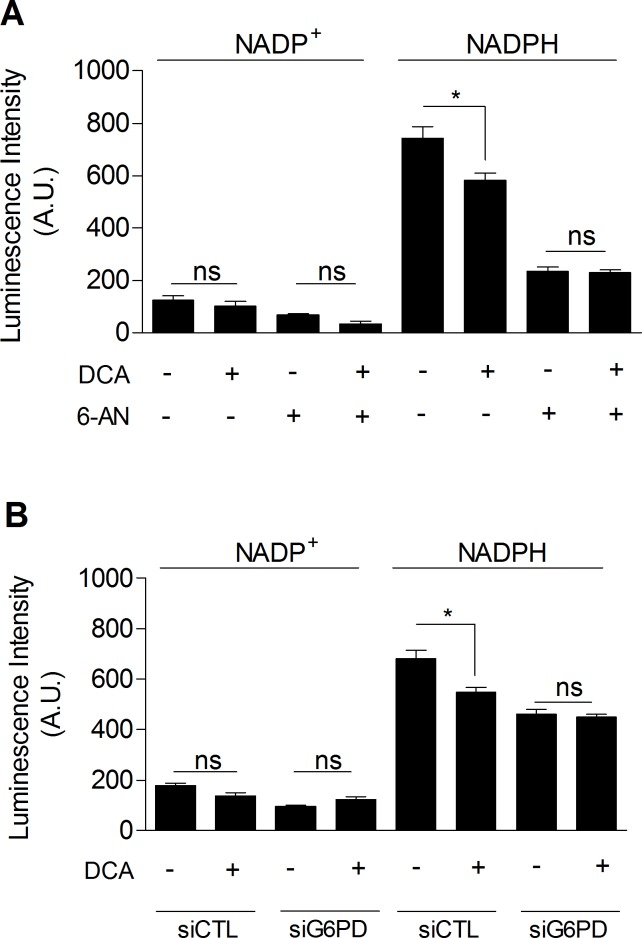
DCA decreases PPP activity **A.** Intracellular NADP^+^ and NADPH levels measured in MDA-MB-231 cancer cells treated with ± DCA (5 mM, 48 h) and ± 6-AN (100 μM, 48 h). **B.** Intracellular NADP^+^ and NADPH levels measured in MDA-MB-231 cancer cells transfected with siRNAs against G6PD (siG6PD) or non-targeting siRNAs (siCTL) and treated with ± DCA. One-way ANOVA with Bonferroni post-hoc test. *p < 0.05, ns, not significant. Results are expressed as means ± SEM.

Finally, we investigated whether DCA could inhibit the PPP, which would explain the link between glycolysis inhibition and the decreased proliferation rate of DCA-treated cancer cells. As shown in Figure 5, we observed that DCA (5 mM, 48 h) significantly decreased NADPH_tot_ level in MDA-MB-231 cancer cells (Figure 5A). Furthermore, while no additional decrease in NADPH_tot_ was obtained when cells were exposed to DCA and 6-AN together (Figure 5A), we showed that DCA specifically decreased NADPH_PPP_. Similar results were found using molecular inhibition of G6PD (Figure 5B). These findings demonstrate the implication of the PPP in the effects of DCA on glycolytic cancer cells.

## DISCUSSION

This study emphasizes the predominant role of the pentose phosphate pathway in cancer biology. The Warburg effect (the fermentation of glucose to lactate in the presence of a physiological O_2_ concentration) observed in numerous cancer cells has been proposed to meet both energetic (ATP) and biosynthetic demands [4, 10]. In this study, we showed that enhanced aerobic glycolysis was not sufficient to maintain ATP levels in cancer cells. Hence, low intracellular ATP level was associated with increased glycolysis. Moreover, the glycolytic switch induced in mitochondria-deficient cancer cells was not able to provide adequate ATP supply as compared to wild-type cells. It is actually suggested that, bioenergetically speaking, a glycolytic metabolism is less efficient than an oxidative one, but that glycolysis may be advantageous to rapidly produce ATP to meet short-timescale energy demands [24]. Our findings also corroborates that Warburg-phenotype cancer cells develop several adaptations to maintain low ATP levels in order to avoid the allosteric inhibition of rate-limiting glycolytic enzymes (the Pasteur Effect) and to keep elevated glycolytic flux rates [10]. On the other hand, we showed that glycolysis promotes cancer cell proliferation, supporting the clinically tested strategies that inhibit the Warburg Effect [12, 25]. The positive relationship between glycolytic efficiency (moles of lactate produced per mole of glucose consumed) and the proliferation rate of cancer cells highlighted that rapidly dividing cells ferment large amounts of glucose in lactate. Recent studies have proposed that lactate production allows cancer cells to efficiently regenerate NAD^+^ by the enzyme lactate dehydrogenase [26]. By maintaining NAD^+^/NADH redox balance, cancer cells allow faster glucose flux through glycolysis, along with faster incorporation of glucose metabolites into biosynthetic pathways, thereby conferring advantages for proliferation [10].

To test whether enhanced aerobic glycolysis promotes cancer cells proliferation by fuelling anabolic processes, involvement of the pentose phosphate pathway (PPP) was evaluated. Indeed, it is known that the PPP uses glucose-6-phophate (the product of the glycolytic enzyme hexokinase) to supply cells with nucleotides and NADPH, a crucial reductant in anabolic reactions [20]. In our study, we showed that PPP flux, evaluated by the NADPH_ppp_/NADP^+^ ratio, was enhanced in Warburg-phenotype as compared to oxidative cancer cells. Comparison of the activity of PPP enzymes could also have been assayed to confirm this difference in PPP flux. Nevertheless, our results support that activation of glycolysis is accompanied by an increase in PPP activity for biosynthesis in rapidly dividing cells [20]. Convincingly, we observed that inhibition of the PPP decreased cancer cell proliferation, with a major effect in Warburg-phenotype cancer cells. The different sensitivity of glycolytic and oxidative cancer cells to PPP inhibition is likely due to the variable reliance on this pathway for macromolecules synthesis; such as lipids and nucleotides. In addition, cancer cells with elevated mitochondrial activity may compensate NADPH levels by using TCA cycle-associated enzymes (malic enzymes and isocitrate dehydrogenases) to replenish the NADPH pool [20]. Taken together, these results demonstrate that cancer cells, especially aggressive Warburg-phenotype cancer cells, rely on the pentose phosphate pathway for optimal proliferation.

To evaluate the therapeutic relevance of these findings, the effects of DCA were investigated. DCA has already shown interesting anticancer properties *in vitro* and *in vivo* [17], and is currently tested in Phase I-II clinical trials [12]. To date, DCA owes its therapeutic properties to the re-sensitization of cancer cells to apoptosis [17]. However, as recently pointed out by Stockwin et al. [27], DCA is relatively inactive on cell viability and induces apoptosis only at high concentrations. Stockwin et al. further reported that DCA was preferentially active in cells with mitochondrial defect. Therefore, we postulated that, at lower dose, DCA also decreases cancer cells proliferation by reducing glycolysis. We found that 5 mM DCA was more effective in Warburg-phenotype cancer cells, reducing cell proliferation by decreasing glycolysis. The different sensitivities among cell lines with different metabolic phenotype may result from the capacity of DCA to reach PDK in the matrix of mitochondria. Indeed, like pyruvate, DCA is ionized and cannot pass through membranes. Curiously, there have been only few reports on the transport of DCA in the cytosol of mammalian cells [28, 29]. Although not yet investigated, the level of expression of mitochondrial pyruvate transporters (MPCs) may also define DCA efficacy. Furthermore, as DCA has a different *K*i for each of the four PDK isoenzymes [30], the variable sensitivity of cancer cells may result from de differential expression of PDKs.

**Figure 6:**
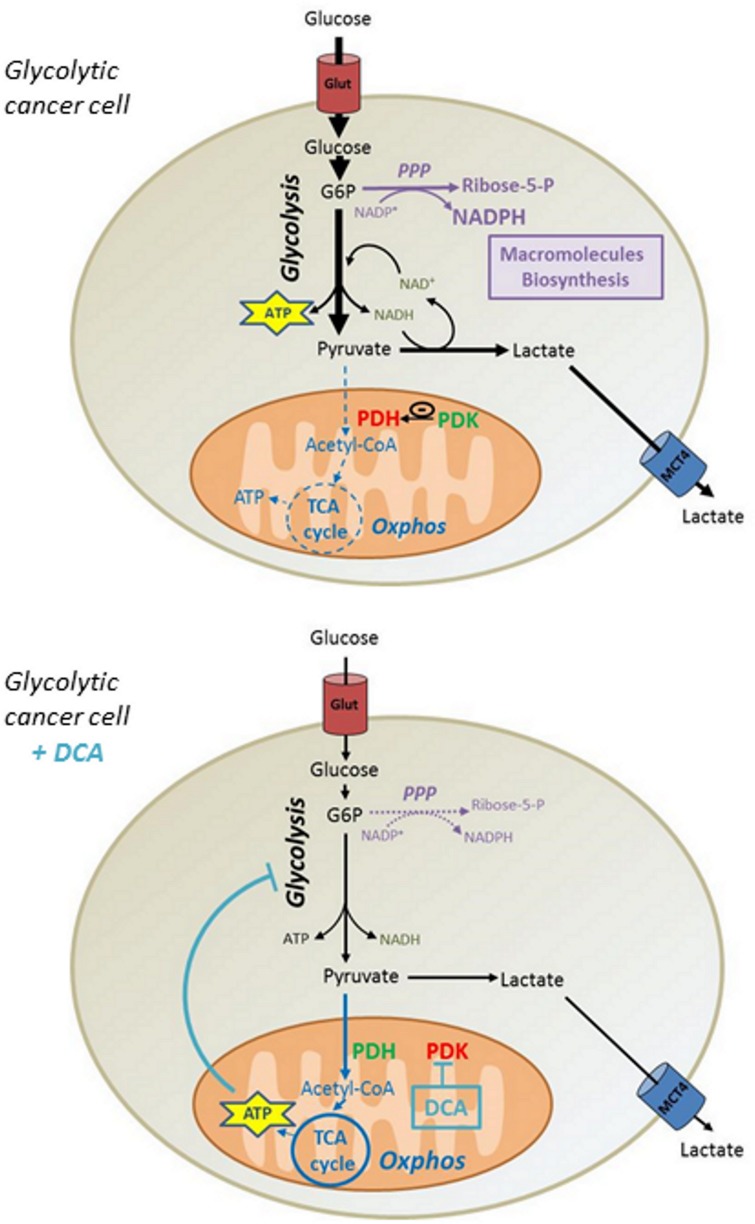
Mechanism by which DCA controls proliferation of glycolytic cancer cells Highly glycolytic cancer cells exhibiting a Warburg phenotype ferment large amounts of glucose into lactate even in the presence of oxygen. This phenomenon allows rapid ATP production and provides precursors for biosynthetic processes promoting cell proliferation. By alleviating PDH inhibition by PDK, DCA fosters the conversion of pyruvate into acetyl-CoA and activates mitochondrial respiration. The consequent allosteric inhibition of glycolysis by ATP reduces glucose consumption and glycolytic intermediates levels, thereby decreasing the flux of the pentose phosphate pathway along with cellular proliferation. PDH, Pyruvate Dehydrogenase. PDK, Pyruvate Dehydrogenase Kinase. G6P, Glucose-6-phosphate. NAD, Nicotinamide Adenine Dinucleotide. NADP, Nicotinamide Adenine Dinucleotide Phosphate. Glut, Glucose Transporter. MCT4, Monocarboxylate Transporter 4.

Although some reports showed that DCA in comparable concentrations promotes cytotoxicity in other cell lines [31, 32], we showed that DCA had no incidence on MDA-MB-231 and SiHa cancer cells viability. Based on our data, we propose that the reactivation of ATP production in mitochondria induced by DCA lowers the glycolytic flux of cancer cells, thereby reducing the incorporation of glucose. Consequently, the decrease in the synthesis of glycolytic intermediates reduces the activity of biosynthetic pathways such as the PPP, compromising the proliferation of Warburg-phenotype cancer cells (Figure 6). Nevertheless, it is conceivable that other mechanisms may participate to the anti-proliferative effects of DCA, as other studies have shown cell cycle arrest at comparable DCA concentrations [33, 34].

Overall, our study showed that the PPP bridges glycolysis and proliferation in aggressive cancer cells, and pleads for considering cancer metabolism for the choice of adequate therapeutic anticancer strategies. In particular, joining the conclusions of others [27], we suggest that clinical development of DCA may benefit from selecting patients with highly glycolytic tumors.

## MATERIALS AND METHODS

### Cell culture and reagents

SiHa human cervix squamous cell carcinoma (ATCC), MDA-MB-231 human breast cancer (ATCC), TLT (transplantable liver tumor) mouse hepatocarcinoma [35], FSaII mouse fibrosarcoma [36], KHT mouse sarcoma [37], NT2 mouse mammary tumor [38] were grown following provider's recommendation or as described. SiHa with partial mitochondrial depletion (ρ0) were obtained by chronic exposure to low concentrations of ethidium bromide as detailed previously [14, 39]. All cultures were kept at 37°C in 5% CO_2_ atmosphere. Cells were incubated in a unique experimental medium 24 hours before the experiments (DMEM without glutamine [Invitrogen], containing 4.5 g/L glucose supplemented with 10 % heat inactivated FBS and 1% penicillin-streptomycin). When SiHa ρ0 were compared to SiHa wild-type, DMEM without glutamine (Invitrogen), containing 4.5 g/L glucose supplemented with 1% pyruvate, 10 % heat-inactivated FBS, 1% penicillin-streptomycin and 50 ng/ml uridine was used. Unless stated otherwise, experiments were performed on confluent cells. Oligomycin in an ATP synthase inhibitor. Dichloroacetate (DCA) is a pyruvate dehydrogenase kinase (PDK) inhibitor. 6-aminonicotinamide (6-AN) is an inhibitor of NADP^+^-dependent enzymes of the pentose phosphate pathway, glucose-6-phosphate dehydrogenase (G6PD) and 6-phosphogluconate dehydrogenase (6-PGD). All chemicals were purchased from Sigma. Oligomycin was dissolved in DMSO. DCA and 6-AN were directly dissolved in culture media.

### siRNA transfection

ON-TARGETplus SMARTpool siRNA against human G6PD and ON-TARGETplus Non-targeting siRNA were from Dharmacon. Final siRNA concentration was 25 nM. All siRNA were transfected using RNAi/MAX according to manufacturer's instructions (Invitrogen).

### Western blotting

Whole cell lysates were collected and subjected to immunoblot analysis as previously described [40]. Primary antibodies were human monoclonals against G6PD or Hsp90 (Sigma).

### Mitochondrial oxygen consumption

The oxygen consumption rate (OCR) of intact whole cells was measured using a Bruker EMX EPR spectrometer operating at 9.5 GHz as previously described [41]. Adherent cells were harvested in fresh experimental medium (10^7^ cells/ml). 100 μl of the cell suspension were mixed with 100 μl of 20% dextran to avoid agglomeration and cells were sealed in a glass capillary tube in the presence of 0.2 mM of a nitroxide probe acting as an oxygen sensor (^15^N 4-oxo-2,2,6,6-tetramethylpiperidine-d_16_-^15^N-1-oxyl, CDN isotopes, Pointe-Claire, Quebec, Canada). Cells were maintained at 37°C during the acquisition of the spectra. EPR linewidth was measured every minute and reported on a calibration curve to obtain the oxygen concentration. OCR was determined by the absolute value of the slope of the decrease in oxygen concentration in the closed capillary tube over time [42]. To obtain OCR from oxidative phosphorylation, OCR in the presence of 1 μM oligomycin treatment was subtracted from the total OCR of the cells.

### Glucose consumption and lactate production

Glucose consumption and lactate production were measured from supernatants of the cultured cells. Metabolite concentrations were quantified on deproteinized samples using specific enzymatic assays on a CMA600 analyzer (CMA Microdialysis AB, Solna, Sweden). Glucose consumption and lactate production were normalized to protein content using the Pierce BCA Protein assay (Thermo Scientific). To efficiently detect differences in metabolite concentrations between SiHa WT and SiHa ρ0 supernatants, a low glucose (1 g/L) medium was used.

### Intracellular ATP quantification

Total intracellular ATP was measured by the ATP Determination Kit (Invitrogen) according to manufacturer's protocol. Cells were washed twice with PBS and lysed in the buffer recommended by the manufacturer (10 mM Tris, 1 mM EDTA, 100 mM NaCl, 0.01% Triton X-100). Cell lysates were added to a reaction mixture containing luciferase and luciferine for bioluminescence measurements using a plate reader (SpectraMax M2e, Molecular Devices). A standard curve was generated with known ATP concentrations in the same conditions. A fraction of the cell lysates was systematically used for protein quantification (Pierce BCA Protein assay, Thermo Scientific) to normalize ATP level to the protein content.

### Proliferation

Cell proliferation was assayed with a 5-bromo-2′-deoxyuridine (BrdU)-ELISA based kit (Roche) following provider's instructions. Sub-confluent cells were incubated in the presence of BrdU (a nucleotide analog) during 4 hours. When proliferation of SiHa WT and SiHa ρ0 were compared, cells were incubated during 6 hours in the presence of BrdU. The amount of BrdU incorporated in the cells was assessed by colorimetric measurements using a plate reader (SpectraMax M2e, Molecular Devices), which allowed the quantitation of DNA synthesis in replicating cells.

### NADPH and NADP^+^ measurements

NADP^+^ and NADPH levels were detected individually from 6,000 harvested cells using a detection kit (NADP/NADPH-Glo Assay, Promega) according to manufacturer's instructions.

### Statistics

All results are expressed as means ± standard error of the mean (SEM). Statistical analyses were performed using the GraphPad Prism 5 software. *P*<0.05 was considered statistically significant.

## SUPPLEMENTARY MATERIAL FIGURES


