# Supplementary material for: Inhibition of the pentose phosphate pathway by dichloroacetate unravels a missing link between aerobic glycolysis and cancer cell proliferation
Source: Oncotarget. 2015 Nov 2;7(3):2910–20. doi: 10.18632/oncotarget.6272 (PMC4823080; doi:10.18632/oncotarget.6272)
Supplement: Supplementary file 1 [file oncotarget-07-2910-s001.pdf]

Inhibition of the pentose phosphate pathway by dichloroacetate unravels a missing link between aerobic glycolysis and cancer cell proliferation

Supplementary Material

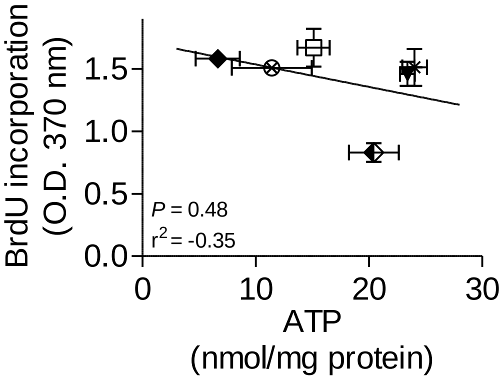

Fig. S1. DNA synthesis is not correlated to intracellular ATP content in cancer cells. Measurements were performed after 24 h incubation in the presence of a culture medium containing only glucose as energetic fuel. Total ATP was quantified from lysed cells and normalized to protein content. Proliferation rates were analyzed by the incorporation of a nucleotid analog (5-bromo-2'-deoxyuridine [BrdU]). A non-significant correlation was found (*p*-value = 0.48, Pearson r = -0.35). Results are expressed as means ± SEM.

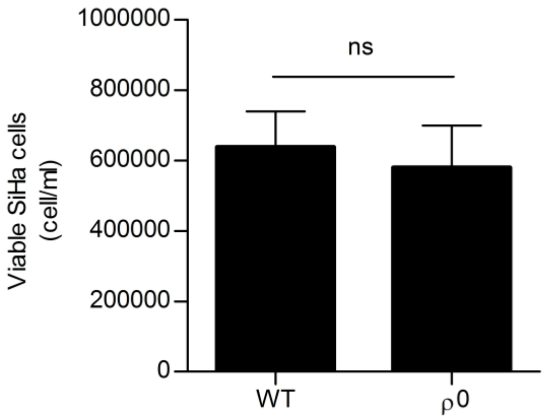

Fig. S2: Viability assays show no difference in the number of viable WT and ρ0 SiHa cancer cells. Viability assays were performed on wild-type (WT) and mitochondria-deficient (ρ0) SiHa cancer cells using trypan blue exclusion after 24 h incubation in the experimental medium (DMEM without glutamine, containing 4.5 g/L glucose supplemented with 10 % heat inactivated FBS and 1% penicillin-streptomycin). Results are expressed as means ± SEM. Two-sided *t* test. ns, non-significant.

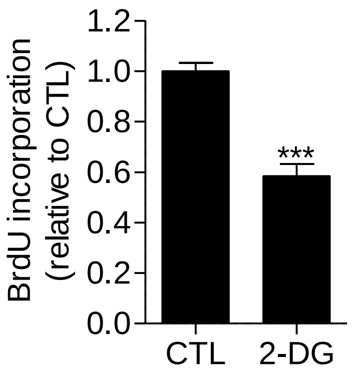

Fig. S3: Treatment with the glycolysis inhibitor 2-Deoxy-D-glucose impairs proliferation of MDA-MB-231 cancer cells. MDA-MB-231 cancer cells were exposed to 5 mM 2-Deoxy-D-glucose (Sigma) during 48 h. Proliferation rates were analyzed by the incorporation of a nucleotid analog (5-bromo-2'-deoxyuridine [BrdU]) incubated during 4 h in the presence of the cells. Two-sided *t* test. ***p<0.001. Results are expressed as means ± SEM.

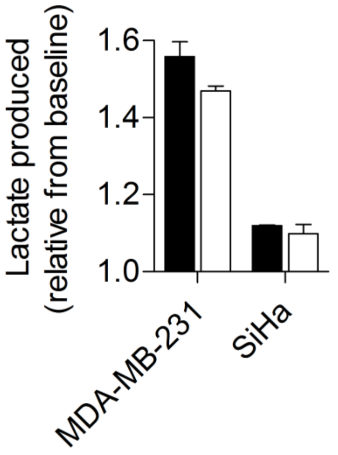

Fig. S4: Short-term lactate production measurements reveal that DCA is more effective in Warburg-phenotype cancer cells. Lactate production by MDA-MB-231 and SiHa cancer cells treated or non-treated with DCA 5 mM during 1 h. Results are expressed as the relative change in lactate concentration from baseline.

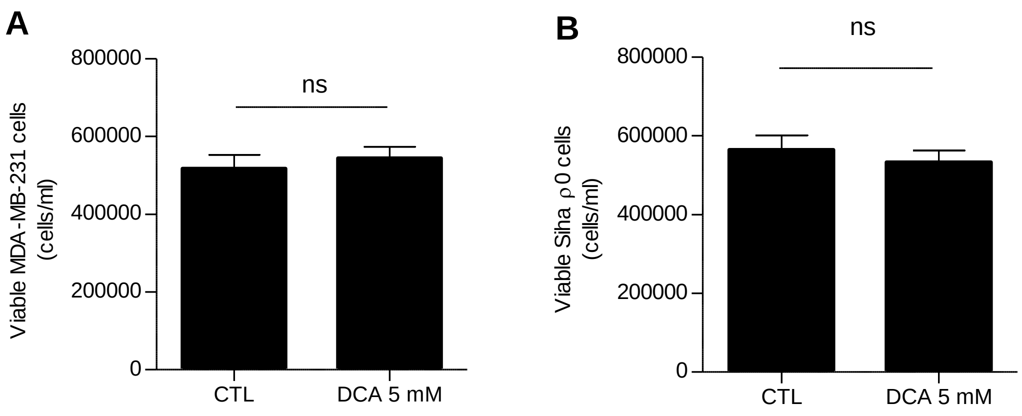

Fig. S5: DCA treatment does not induce cancer cell death. Viability assays using trypan blue exclusion were performed on MDA-MB-231 cancer cells (A) and SiHa ρ0 cancer cells (B) treated or non-treated with DCA 5 mM during 48 h. The absence of significant difference in viable cell number between treated and non-treated cells indicated that cell mortality was not induced by DCA treatment. Results are expressed as means ± SEM. Two-sided *t* test. ns, non-significant.

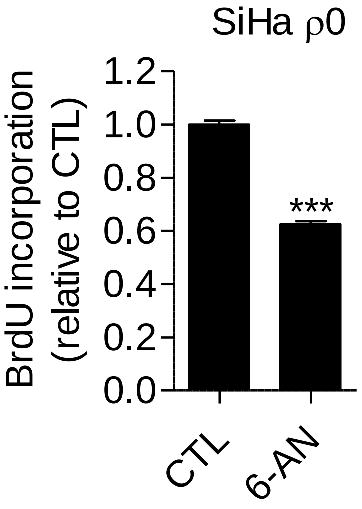

Fig. S6: Treatment with 6-AN impairs proliferation of SiHa ρ0 cancer cells. SiHa ρ0 cancer cells were exposed to 100 µM 6-AN during 48 h. Proliferation rates were analyzed by the incorporation of a nucleotid analog (5-bromo-2'-deoxyuridine [BrdU]) incubated during 4 h in the presence of the cells. Two-sided *t* test. ****p*<0.001. Results are expressed as means ± SEM.
